# Advillin is a tuft cell marker in the mouse alimentary tract

**DOI:** 10.1007/s10735-020-09893-6

**Published:** 2020-07-02

**Authors:** Anna-Lena Ruppert, Maryam Keshavarz, Sarah Winterberg, Johannes Oberwinkler, Wolfgang Kummer, Burkhard Schütz

**Affiliations:** 1 Institute for Anatomy and Cell Biology, Philipps-University, Robert-Koch-Straße 8, 35037 Marburg, Germany; 2 Institute for Anatomy and Cell Biology, Justus-Liebig-University, Aulweg 123, 35385 Gießen, Germany; 3 Institute for Physiology and Pathophysiology, Philipps-University, Deutschhausstraße 1, 35037 Marburg, Germany

**Keywords:** Doublecortin-like kinase 1, Intestine, Biliary tract, Immunohistochemistry, In situ hybridization, Villin

## Abstract

Tuft cells are a rare population of chemosensory cells at the mucosal surface epithelia of hollow organs. Their name-giving morphological feature is an apical tuft of stiff microvilli. Accordingly, the actin-binding protein, villin, was identified as one of the first tuft cell markers in immunohistochemical analysis. Unfortunately, villin expression is not restricted to tuft cells, but is also prominent e.g. in enterocytes, which limits the use of this gene as a marker and as an experimental tool to genetically target tuft cells. Here, we report that the villin-related protein, advillin, is a specific tuft cell marker in the gastro-intestinal and biliary tract epithelia. In situ hybridization and immunohistochemistry revealed that advillin expression, unlike villin, was restricted to solitary cholinergic tuft cells in the mucosal linings of the small and large intestine, and in the gall bladder. In the glandular stomach, villin and advillin mRNA were present in all epithelial cells, while detectable protein levels were confined to solitary tuft cells. Advillin expression was no longer detectable in the mucosa of the intestinal and biliary tract from *Pou2f3* deficient mice that lack tuft cells. Finally, crossing *Avil-Cre* transgenic mice with a double-fluorescent reporter mouse line resulted in specific targeting of gastro-intestinal and biliary tuft cells. Our analysis introduces advillin as a selective marker and tool in histological and functional analysis of the alimentary tract tuft cell system.

**Electronic supplementary material:**

The online version of this article (doi:10.1007/s10735-020-09893-6) contains supplementary material, which is available to authorized users.

## Introduction

Tuft cells, also known as brush cells, are columnar, often flask-shaped cells, and found scattered in the simple surface epithelia of endoderm-derived hollow organs. Originally, they were identified in an electron microscopic analysis of the rat trachea (Rhodin and Dalhamn 1956), and subsequently also observed in intestine and gall bladder (Luciano and Reale 1969; Trier et al. 1987). A characteristic, and hence name-giving morphological feature is the presence of an apical tuft of thick and straight microvilli, that reaches into the lumen of the hollow organ. These microvilli contain axial bundles of actin filaments with elongated rootlets of cytoplasmic filaments, which often terminate close to the cell nucleus.

In the 1990s, immunohistochemistry with antibodies against villin and fimbrin, two actin filament crosslinking proteins, led to an intense staining of the tuft cell apex in the rat major pancreatic duct, submandibular gland, trachea, and intestine (Höfer and Drenckhahn 1992). Although the use of villin-antibodies allowed unequivocal identification of tuft cells in the first three of these sites, in the intestine the whole enterocyte brush border was labeled, making antibodies against villin not well suited as a selective tuft cell marker at this location (Höfer and Drenckhahn 1992). Also, other structural marker proteins, like cytokeratin-18 and β-tubulin, were found highly, but not selectively expressed by tuft cells (Bezençon et al. 2008; Höfer and Drenckhahn 1996; Schütz et al. 2015, 2019).

A major advancement was reached when tuft cells were found to share molecular features with taste cells in the oral cavity. Several members of the taste transduction pathway, e.g. α-gustducin (Höfer et al. 1996), phospholipase C isoform β2 (Bezençon et al. 2007), and transient receptor potential cation channel subfamily M member 5 (TRPM5) (Kaske et al. 2007) were identified in gastro-intestinal tuft cells, suggesting a function of this cell type in chemoreception (Finger and Kinnamon 2011). Microarray and RT-PCR analysis of small intestinal cell fractions enriched in tuft cells from mice expressing enhanced green fluorescent protein (EGFP) under the control of the *Trpm5* promoter (Bezençon et al. 2007) subsequently uncovered another structural marker, advillin, whose expression was found to be restricted to tuft cells (Bezençon et al. 2008).

Advillin, initially named p92 (Marks et al. 1998), is a member of the gelsolin/villin superfamily of actin regulatory proteins. Advillin expression pattern analysis suggested that it is expressed almost exclusively by sensory neurons during development and in adulthood (Chuang et al. 2018; Hasegawa et al. 2007). Recently, *Avil* promoter-driven expression of either *Egfp* or *Cre*-recombinase extended the *Avil* expression pattern to diverse sets of central and peripheral neurons, including all neural crest-derived ganglia (Hunter et al. 2018). In another mouse line expressing *Cre*-recombinase under the control of the *Avil* promoter, also taste buds in the circumvallate papilla stained positive (Zurborg et al. 2011).

Since mouse advillin and villin share about 59% identity on the protein level (www.ensembl.org), it was speculated that published immunohistochemical staining of tuft cells with anti-villin antibodies did not represent true expression, but resulted from cross-reactivity with advillin (Bezençon et al. 2008). Together with the reported tuft cell-specific presence of *Avil* mRNA in the mouse small intestine (Bezençon et al. 2008), this led us to compare the expression profiles of villin and advillin in the mouse gastro-intestinal and extra-hepatic biliary tract on a cellular level in tissue sections using sensitive in situ hybridization and immunohistochemistry. In addition, the suitability of the afore-mentioned *Avil*-*Cre* mouse line to specifically target gastro-intestinal and biliary tuft cells was investigated.

## Materials and methods

### Mouse strains and animal procedures

Bacterial artificial chromosome-transgenic mice that express EGFP under the control of the choline acetyltransferase (*Chat*) promoter (Schütz et al. 2015; von Engelhardt et al. 2007) were obtained from in house breeding. POU domain, class 2, transcription factor 3 (*Pou2f3*)-deficient mice (named *Pou2f3*^−/−^ throughout the study) (Matsumoto et al. 2011) were obtained from the Monell Chemical Senses Center (Philadelphia, PA, USA) and maintained in house. A double-fluorescent Cre reporter mouse line, mT/mG (Muzumdar et al. 2007), was obtained from JaxMice (B6.129(Cg)-*Gt(ROSA)26Sor*^*tm4(ACTB−tdTomato,−EGFP)Luo*^/J, stock no. 007676, The Jackson Laboratory, Bar Harbor, ME, USA). A mouse line that expresses Cre-recombinase under the control of the *Avil* promoter (*Avil*-Cre, European Mouse Mutant Archive repository, Id EM:05542; Infrafrontier GmbH, Neuherberg, Germany) (Zurborg et al. 2011) was obtained from the Institute for Pharmacology, University of Heidelberg (Heidelberg, Germany). All mice were housed in groups of 3–6 under specified pathogen-free conditions. They were kept on a 12 h light/12 h dark cycle and had access to food and water ad libitum. The genotypes of all mice were verified by PCR using genomic DNA obtained from ear biopsies, according to published protocols supplied by the vendors (The Jackson Laboratory) or donators (H. Monyer, Heidelberg, Germany, for ChAT-EGFP mice; I. Matsumoto, Tokyo, Japan, for Pou2f3 mice; P. Heppenstall, Monterotondo, Italy, for Avil-Cre mice). All animal procedures were conducted in accordance with EU Directive 2010/63/EU for animal experiments, the German Animal Protection Law, and received methodological and ethical approval by the local animal welfare officer (protocols: Ex-15-2016, Ex-14-2018, 641-M). A minimum of three mice of both sexes at 12–16 weeks of age were used for all analyses.

### Tissue harvesting for histological analysis

All mice were sedated by inhalation of isoflurane (Forene, order-no. B506, AbbVie Deutschland GmbH & Co. KG, Wiesbaden, Germany) and sacrificed by cervical dislocation. For the visualization of native EGFP fluorescence, organs were dissected, cut open, mounted flat in a droplet of 0.9% saline (2350748, B. Braun Melsungen AG, Melsungen, Germany) on a microscopic slide, and cover-slipped. For in situ hybridization (ISH) and immunohistochemistry (IHC), the vallate papilla was identified at the back of the tongue and removed with surrounding muscle tissue. The stomach was opened along the large curvature and the content washed out. The gall bladder was left attached to pieces of surrounding liver tissue. Tissue pieces from duodenum (including pancreas), jejunum, ileum and colon, all 0.5–1 cm in length, were quickly dissected. Lumbar dorsal root ganglia were identified lateral from the spinal cord and dissected after opening the spine from the dorsal side caudal of the 12th rib. The tissues used for ISH were submerged in TissueTek compound (4583, Sakura Finetek Europe B.V, Alphen aan den Rijn, The Netherlands) and quickly frozen in isopentane (2-Methylbutane, M32631, Sigma-Aldrich, Steinheim, Germany) at − 40 °C. After an initial storage at − 70 °C, 16 µm thick tissue sections were cut at − 15 °C using a cryostat (CM3050S, Leica Microsystems GmbH, Nussloch, Germany) and mounted on silanized (3-(Triethoxysilyl)-propylamine, 8.21619.0100, Merck Millipore, Darmstadt, Germany) glass slides. Tissue used for immunohistochemistry was processed in two ways: Some samples were immersion-fixed in Bouin Hollande fixative, then extensively washed in 70% isopropanol (9866.4, Carl Roth GmbH + Co. KG, Karlsruhe, Germany), dehydrated, and embedded in paraffin (Tissue-Tek Paraffin Wax, 4523, Sakura Finetek) (Schütz et al. 2008). Paraffin blocks were cut with a microtome (Microm HM 325, Thermo Scientific, Schwerte, Germany) and 7 µm thick sections were mounted on silanized microscope glass slides. Alternatively, tissues were fixed in 4% buffered (pH 7.4) paraformaldehyde (P6148, Sigma-Aldrich), cryo-protected with 20% sucrose (9097, Carl Roth), frozen in isopentane, and 16 µm sections cut with a cryostat.

### In situ hybridization

Tissue sections on glass slides were air-dried at room temperature for 30 min and subjected to the following procedure (all procedures at room temperature unless otherwise stated): 1 h fixation in freshly prepared 4% (w/v) paraformaldehyde (P6148, Sigma-Aldrich) in phosphate-buffered saline (PBS, pH 7.5 (10 X: 77 mM Na_2_HPO_4_ (N350.1, Carl Roth), 23 mM NaH_2_PO_4_ (K300.1, Carl Roth), 1,53 M NaCl (146994.1214, AppliChem, Darmstadt, Germany)), three times 10 min each washing in PBS, permeabilization for 10 min in 0.4% (v/v) Triton X100 (3051.3, Carl Roth) in PBS. After additional washes in PBS, the sections were acetylated for 10 min with triethanolamine (T58300, Sigma-Aldrich)/acetic anhydride (320102, Sigma-Aldrich), washed again, dehydrated in 50% and 70% isopropanol, and finally air-dried. Complementary RNA probes for the detection of mouse *Vil* and *Avil* transcripts in tissue sections were generated from mouse C57BL/6 ileum cDNA. For *Vil*, a 788 nt long DNA fragment (GeneBank acc. no. NM_009509.2, nt 1521-2318) was amplified by PCR, and subcloned into pGEM-T (pGEM-T Vector System, A3600, Promega, Mannheim, Germany). For *Avil*, a 891 nt long DNA fragment (GeneBank acc. no. NM_009635.3, nt 1804-2694) was amplified by PCR, and subcloned into pGEM-T. For the detection of *Egfp* transcripts, a 601 bp fragment from the EGFP coding sequence (pEGFP-N1, Clontech, Palo Alto, USA) was used (Schütz et al. 2015). The identity of the cloned gene fragments was confirmed by double-stranded sequencing (Microsynth Seqlab GmbH, Göttingen, Germany). Antisense and sense riboprobes were generated by in vitro transcription using T7 (T7 RNA Polymerase, R0884, Sigma-Aldrich) and SP6 polymerase (SP6 RNA Polymerase, 11487671001, Roche Diagnostics, Mannheim, Germany), respectively, and radioactive (UTPαS, [35S], NEG039C001MC, PerkinElmer, Waltham, USA), or non-radioactive (digoxygenin-11-UTP, DIG RNA Labeling Mix, 11277073910, Roche Diagnostics)-labeled nucleotides. The ISH procedure was essentially performed as described previously (Schütz et al. 2015), with a few modifications. The tissue sections on microscopic slides were covered with 30–40 μl of hybridization solution, containing 50% formamide (24311.291, VWR International S.A.S, Briare, France), 0.6 M NaCl (146994.1214, AppliChem), 10 mM Tris/HCl (pH 7.4) (Tris–HCL, 9090.3, and Tris-Base, 4855.2, Carl Roth), 1 mM Na_2_EDTA (8043.2, Carl Roth), 1 X Denhardt’s (Denhardt´s Solution, D9905, Sigma-Aldrich), 10% dextran sulfate (dextran sulfate sodium salt from *Leuconostoc* spp., 31395, Sigma-Aldrich), 100 µg/ml sonicated salmon sperm DNA (sonicated salmon sperm DNA, 201190, Agilent, Santa Clara, USA), 0.05% (w/v) *E. coli* MRE600 tRNA (10109550001, Sigma-Aldrich), 20 mM dithiothreitol (DTT) (90469024, Roche), 50,000 d.p.m./µl S35-labeled riboprobe, 2 ng/µl digoxygenin-labeled riboprobe (when performing double-probe experiments), and cover-slipped. Hybridization was carried out overnight at 60 °C in a humid chamber (Nunc Square BioAssay Dishes, 240835, Thermo Scientific). After hybridization, coverslips were removed in 2 X standard saline/sodium citrate (SSC) (20 X: 3 M NaCl (146994.1214, AppliChem), 0,3 M Na_3_citrate × 2 H2O (3580.1, Carl Roth), 5 M HCl (9277.1, Carl Roth)) and the sections washed in the following order: 15 min in 2 X SSC, 15 min in 1 X SSC, 30 min at 37 °C in RNase solution (20 µg/ml RNase A (A3832.0500, AppliChem) and 1 U/ml RNase T1 (10109193001, Roche)), 30 min at RT in RNase-solution, 15 min in 1 X SSC, 15 min in 0.5 X SSC, 15 min in 0.2 X SSC, 60 min at 60 °C in 0.2 X SSC, 15 min in 0.2 X SSC, and finally 15 min in distilled water. The detection of digoxygenin-labeled *Egfp* probes was performed with alkaline phosphatase (AP)-conjugated anti-digoxygenin antibodies (11093274910, Anti-digoxigenin-AP, Fab fragments, Roche) diluted to 1.5 U/ml, and 0.2 mM BCIP (5-Bromo-4-chloro-3-indolyl phosphate, 4-toluidine salt, 11383221001, Roche) and NBT (4-nitro blue tetrazolium chloride solution, 11383213001, Roche) using the manufacturers protocol, which yielded a purple-blue precipitate after 4–6 h. For the visualization of radioactive hybridization signals, sections were exposed to Carestream BIOMAX MR autoradiography film (Z358460-50EA, Sigma-Aldrich) for 2–3 days to estimate further exposure times, then coated under absence of light with nuclear emulsion (Ilford K5, 1355136, Harman Tech., Mobberley, UK), exposed for 2 weeks at 4 °C in the dark and finally developed using Ilford Phenisol developer (1757635, Harman) and Ilford Hypam fixer (1758285, Harman). Sections were counterstained with methyl green (323829, Sigma-Aldrich), and cover-slipped. Antisense and sense RNA probes were run in parallel in the same experiment to ensure equivalent conditions. In each experiment, vallate papilla containing taste buds was used as positive control.

### Immunohistochemistry

Single brightfield IHC was performed as described previously (Schütz et al. 2015). All procedures were done at room temperature unless otherwise stated. Paraffin-embedded tissue sections were deparaffinized in xylene (4436.2, Carl Roth) and rehydrated through a graded series of isopropanol (6752.5, Carl Roth), including 30 min incubation in methanol/0.3% H_2_O_2_ (methanol, 4627.5, Carl Roth; hydrogen peroxide 30%, 8070.2, Carl Roth) to block endogenous peroxidase activity. Subsequently, antigen retrieval was achieved by incubation in 10 mM sodium citrate buffer (0.1 M citric acid monohydrate, 1.00244.1000, Merck Millipore; 0.1 M tri-sodium citrate dihydrate, 3580.3, Carl Roth; pH 6.0) at 92–95 °C for 10 min. Frozen sections were thawed and dried at 42 °C for 45 min. For permeabilization, the sections were incubated for 10 min in 0.4% Triton X100 (3051.3, Carl Roth) followed by a 30 min incubation in 0.3% H_2_O_2_ (80,702, Carl Roth) to block endogenous peroxidase activity.

Non-specific binding sites were blocked with 5% bovine serum albumin (BSA) (albumin Fraction V, 8076.3, Carl Roth) in 50 mM PBS for 30 min, followed by an avidin–biotin blocking step (Avidin–Biotin Blocking Kit, SP-2001, Vector Laboratories, Burlingame, USA) for 20 min each. Primary antibodies included: rabbit anti-human advillin (ABIN4278537, antibodies-online.de, Aachen, Germany; 1:200 final dilution), mouse anti-human villin (sc-365310, Santa Cruz Biotech. Inc., Heidelberg, Germany; 1:200), chicken anti-EGFP (NB100-1614, Bio-Techne GmbH, Wiesbaden, Germany; 1:5,000). Primary antibodies were applied in PBS/1% BSA over night at 16 °C followed by 2 h at 37 °C. After 3 × 5 min washes in double-distilled water and 10 min in PBS, the sections were incubated for 45 min at 37 °C with species-specific biotinylated secondary antibodies (biotin-SP AffiniPure donkey anti-rabbit IgG (H + L), 711-065-152; biotin-SP AffiniPure donkey anti-mouse IgG (H + L), 715-065-151; biotin-SP AffiniPure donkey anti-chicken IgY (IgG) (H + L), 703-065-155; all from Jackson ImmunoResearch, Ely, UK), diluted 1:200 in PBS/1% BSA, washed, and incubated for 30 min with avidin–biotin–peroxidase complex (Vectastain Elite ABC kit; PK-6100, Vector Laboratories, Burlingame, USA). Immunoreactions were then visualized by 8 min incubation in 100 mg/16 ml DAB (3,3′-diaminobenzidine tetrahydrochloride hydrate, D5637, Sigma-Aldrich), enhanced by the addition of 600 mg/8 ml ammonium nickel (II) sulfate hexahydrate (098825, Sigma-Aldrich). After three 5 min washes in distilled water, sections were counterstained with hemalaun solution (Hämatoxylin cryst., 104302, Merck Millipore), dehydrated through a graded series of isopropanol, cleared in xylene and finally mounted under coverslips. Digital brightfield pictures were taken with an Olympus AX70 microscope (Olympus Optical, Hamburg, Germany), equipped with an Olympus UC90 camera and Olympus cellSens analyses software.

For double brightfield IHC with two primary antibodies from the same donor species, i.e. rabbit anti-human advillin and rabbit-anti-human Doublecortin-like kinase 1 (DCLK1, AP7219b, Abgent, San Diego, CA, USA; 1:100), sections were sequentially stained in the following way: staining with the anti-advillin antiserum was done as described above, using DAB with nickel enhancement, which resulted in dark blue/black reaction product. After dehydration and again rehydration, staining with the anti-DCLK1 antiserum was visualized with DAB without addition of nickel, which resulted in a brownish staining. Advillin/DCLK1 co-immunoreactive cells thus should display both colors, although mostly in separate intracellular compartments, while additional single DCLK1-immunoreactive cells should stain brownish only.

Double immunofluorescence analysis was performed on paraffin sections as follows: After deparaffinization and blocking procedures (see above), anti-EGFP (1:500 final dilution) and anti-advillin (1:20) were co-applied in PBS/1% BSA and incubated over night at 16 °C, followed by 2 h at 37 °C. After extensive washing in distilled water followed by PBS, immunoreactions for AVIL were visualized with a Cy^TM^3 AffiniPure donkey anti-rabbit IgG (H + L) antibody (711-165-152; 1:100, Jackson ImmunoResearch). EGFP immunoreactions were visualized by a two-step procedure using a biotin-SP AffiniPure donkey anti-chicken IgY (H + L) secondary antibody (703–065-155, 1:200, Jackson ImmunoResearch), followed by streptavidin, Alexa Fluor™ 488 conjugate (S11223, 1:200, Invitrogen). Incubation times were 45 min with the biotinylated secondary antibody only, followed by 2 h incubation with a mixture of fluorochrome-conjugated secondary antibody and streptavidin. Immunofluorescence signals were documented with a Zeiss Imager M2 microscope (Zeiss, Oberkochen, Germany), equipped with a Zeiss AxioCam HRc camera, and ZEN 2011 software. Co-expression ratios of two labels were determined by visual examination of 4–6 tissue sections each derived from 3 mice.

Double immunofluorescence analysis of *Avil*-Cre:mT/mG mice was performed on cryosections as follows: After equilibration of the dried sections in PBS, they were permeabilized by incubation for 10 min in 0.4% Triton X100. After brief washes in PBS and distilled water, the sections were submerged for 3 min in methanol. This step eliminated the reporter mouse-intrinsic green (EGFP) and red (tdTomato) fluorescence and allowed the immunofluorescent visualization of two antigens. The subsequent staining procedure was performed as described above, using anti-EGFP (1:500) and anti-DCLK1 (1:20) antisera. Co-expression ratios of EGFP and DCLK1 were determined by visual examination of tissue sections derived from 4 mice.

## Results

### Villin and advillin expression patterns in taste cells and in sensory neurons

**Fig. 1:**
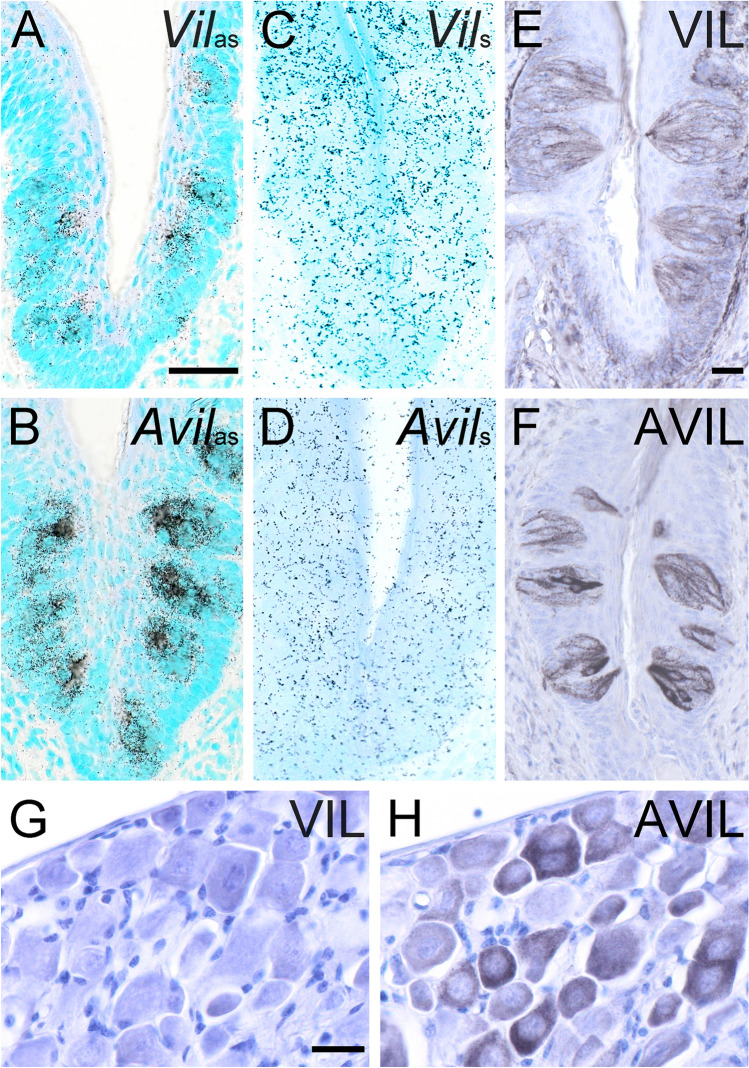
Villin and advillin expression patterns in mouse taste buds and in dorsal root ganglia. *Vil* mRNA (**a**), and *Avil* mRNA (**b**), where both detected with antisense (as) riboprobes in radioactive ISH in taste buds of the mouse vallate papilla. The use of sense (s) riboprobes for *Vil* (**c**) and *Avil* (**d**) did not result in specific staining. Villin-immunoreactivity outlined many, if not all taste cells, with the apical tip showing the most intense staining (**e**). Likewise, advillin-immunoreactive cells were present in taste buds, sometimes showing intense intra-cellular staining (**f**). Absence of villin-immunoreactivity (**g**), and presence of advillin-immunoreactivity (**h**) in dorsal root ganglion neurons. Sections from ISH were counter-stained with methyl green, sections from IHC with hemalaun. The bar in **a** represents 50 µm and applies to **a**–**d**. The bar in **e** represents 20 µm and also applies to **f**. The bar in **g** represents 20 µm and also applies to **h**

For an unambiguous detection of *Vil* and *Avil* mRNA on a cellular level in tissue sections, we designed riboprobes for ISH experiments, and tested these tools on sections containing oral taste buds. Both *Vil* (Fig. 1a) and *Avil* (Fig. 1b) antisense riboprobes selectively labeled taste buds in the mouse vallate papilla, while their corresponding sense riboprobes did not result in specific staining (Fig. 1c, d). On the protein level, villin-immunoreactive taste cells were detected with a monoclonal mouse anti-human villin antibody (Fig. 1e). A similar staining pattern was obtained with a polyclonal rabbit anti-human advillin antiserum (Fig. 1f). Dorsal root ganglia harbor sensory neurons that are known to express *Avil*, but not *Vil* (Chuang et al. 2018). In our hand, the anti-villin antibodies did not show cross-reactivity with mouse advillin, because they failed to label dorsal root ganglia neurons (Fig. 1g), while many neurons were stained with the anti-advillin antibodies (Fig. 1h). Likewise, the anti-advillin antibodies did not display villin cross- reactivity (see below, Figs. 3, 4). Taken together, specific and sensitive detection methods for both, *Vil* and *Avil* transcripts and their protein products on mouse tissue sections could be established.

### Villin and advillin expression patterns in the mouse gastro-intestinal tract

**Fig. 2:**
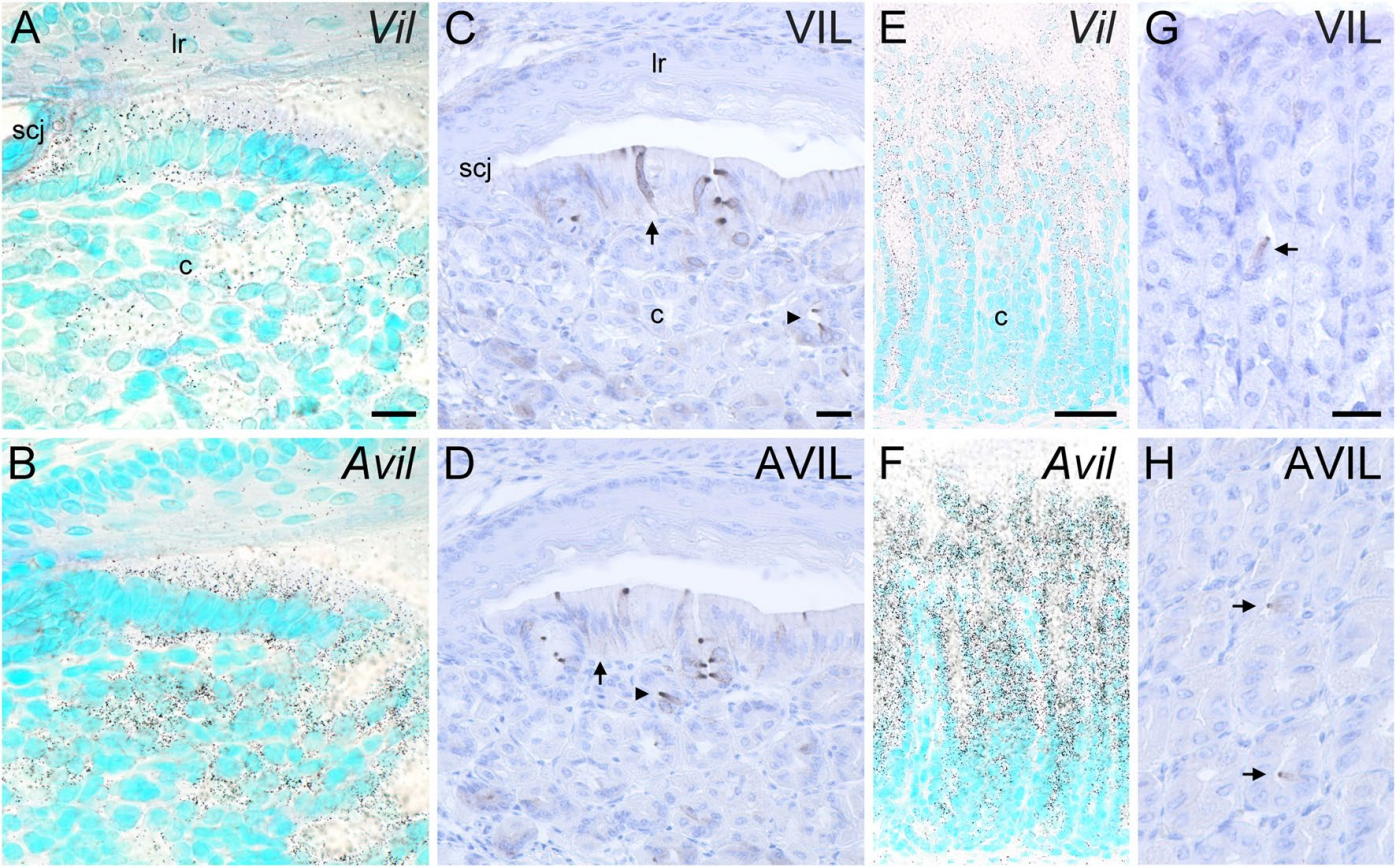
Villin and advillin expression patterns in the mouse stomach. In the mouse stomach, the squamo-columnar junction (scj) represents the histological transition from the limiting ridge (lr) to the corpus (c). (**a**) *Vil* mRNA, detected by radioactive ISH, was present both in epithelial cells of the columnar aspect of the corpus, and in deeper glands. (**b**) *Avil* mRNA displayed a similar expression pattern, with no preference in staining intensity for individual cells. On the protein level, both villin- (**c**) and advillin- (**d**) immunoreactivities were confined to individual cells of the columnar epithelium (arrows) and in deeper glandular aspects (arrowheads). Especially the staining of the apical tips of the columnar and flask-shaped cells was most intense. *Vil* (**e**) and *Avil* (**f**) mRNA was present throughout the mucosal epithelium of the corpus, while immunoreactivity was confined to individual cells (arrows in **g** and **h**). Sections from ISH were counter-stained with methyl green, sections from IHC with hemalaun. The bar in **a** represents 20 µm and also applies to **b**. The bar in **c** represents 20 µm and also applies to **d**. The bar in **e** represents 50 µm and also applies to **f**. The bar in **g** represents 20 µm and also applies to **h**

The mouse stomach is divided into the non-glandular fundus and the glandular corpus, with the limiting ridge representing the anatomical border. At the so-called squamo-columnar junction, the stratified squamous epithelium of the fundus is replaced by a columnar arrangement of epithelial cells of the corpus. Here, clusters of tuft cells that express taste-cell characteristics (Eberle et al. 2013; Hass et al. 2007) are arranged in a palisade-like manner (Luciano and Reale 1992). Using ISH we found that both, *Vil* (Fig. 2a) and *Avil* (Fig. 2b) mRNAs were uniformly expressed in the entire surface epithelium and in the deeper glandular zone, showing no preference for individual cells. *Avil* signal intensities were found generally stronger than those for *Vil*, and the corresponding sense riboprobes did not produce detectable signals (data not shown). On the protein level, villin- (Fig. 2c) and advillin- (Fig. 2d) immunoreactivities were most prominent at the apical tips of individual cells, but also labeled the columnar and flask-shaped cells throughout in a punctate fashion. In all other parts of the corpus, down to the transition between the pylorus and the duodenum, *Vil* (Fig. 2e) and *Avil* (Fig. 2f) mRNAs likewise were uniformly present in the whole surface epithelium, with signal intensities diminishing in deeper glandular aspects. Again, immunoreactivities for villin (Fig. 2g) and advillin (Fig. 2h) were restricted to individual cells. Taken together, on the mRNA level both *Vil* and *Avil* genes were found to be expressed by seemingly all epithelial cells of the glandular stomach, while detectable protein levels were confined to individual cells when using IHC.

**Fig. 3:**
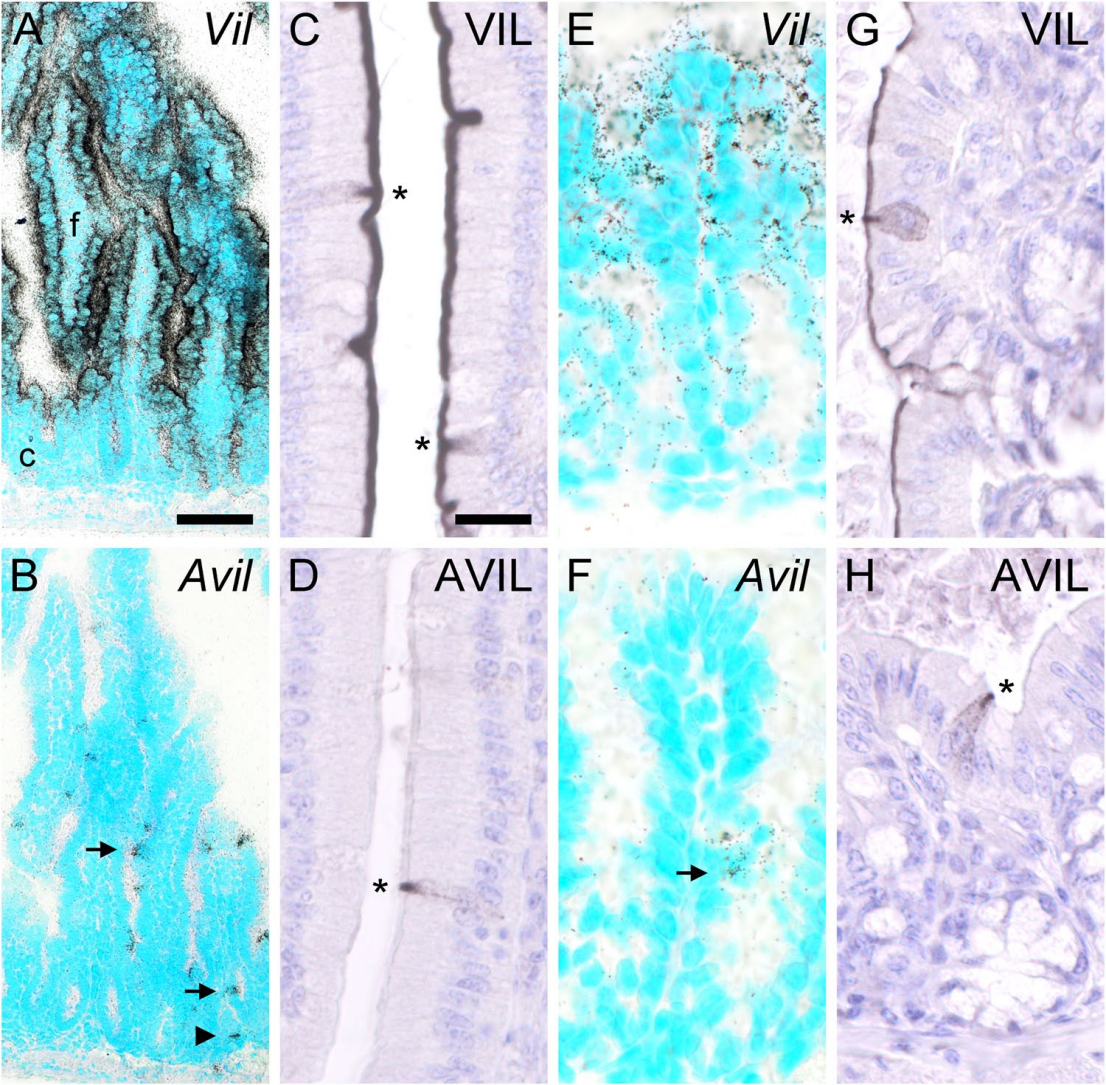
Villin and advillin expression patterns in the mouse small and large intestine. (**a**) *Vil* mRNA, detected with antisense riboprobes in radioactive ISH, was present in the entire epithelial lining of the small intestine, shown here for duodenum. Note much stronger expression in folds (f) compared to crypts (c). (**b**) *Avil* mRNA was confined to individual epithelial cells, both in folds (arrow) and in crypts (arrowhead). (**c**) Villin-immunoreactivity was seen along the entire apical aspects of the duodenal mucosa, occasionally with additional cell body labeling (asterisks). (**d**) Advillin-immunoreactivity was confined to individual cells in the mucosal epithelium. Note prominent staining of the apical tip (asterisk). (**e**) In the large intestine, *Vil* mRNA was mainly present in the upper half of the crypts, shown here for the colon. (**f**) Again, presence of *Avil* mRNA was confined to individual cells. (**g**) Villin-immunoreactivity was seen along the entire apical aspects of the colonic mucosa, occasionally with additional cell body labeling (asterisk). (**h**) Advillin-immunoreactivity was confined to individual cells in the mucosal epithelium (asterisk). Sections from ISH were counter-stained with methyl green, sections from IHC with hemalaun. The bar in **a** represents 100 µm and also applies to **b**. The bar in **c** represents 20 µm and also applies to **d**–**h**

In the rodent small intestine, solitary tuft cells locate to crypts and folds of the single-layered mucosal epithelium and make up approx. 1–7% of all epithelial cells, depending on the microbial status (Howitt et al. 2016). Strong *Vil* ISH signals were present on seemingly all epithelial cells lining the folds, but signals were weak to undetectable in crypts (Fig. 3a). ISH signals for *Avil*, on the other hand, were present on individual cells in both crypts and folds (Fig. 3b). Villin-immunoreactivity strongly labeled the apical brush border of the entire epithelium, with only goblet cells showing weaker staining (Fig. 3c). In some cells, strong immunoreactivity was also seen extending from the luminal side into the cell body, and the antibodies also labeled the whole cell in a punctuate fashion. Advillin-immunoreactivity was confined to individual cells (Fig. 3d). Again, the apical tip showed the most intense labeling, while the rest of the cell displayed punctuate immunoreactivity.

The mucosa of the large intestine is characterized by the presence of straight crypts and the lack of villi. Moderate *Vil* ISH signals were most prominent in the upper half of the crypts and weak to absent in the lower half (Fig. 3e). Again, no preference for individual cells was observable. ISH signals representing *Avil* gene expression again were confined to solitary cells (Fig. 3f). In analogy to the small intestine, villin-immunoreactivity labeled the entire brush border and additionally some solitary cells (Fig. 3g). Advillin-immunoreactivity was found restricted to solitary cells, with staining characteristics similar to that found in the small intestine (Fig. 2h). Taken together, while villin was found expressed by almost all intestinal mucosal epithelial cells, advillin expression was found confined to individual cells, both on the mRNA and on the protein level, suggesting restricted expression in tuft cells.

### Villin and advillin epression patterns in the mouse gall bladder

**Fig. 4:**
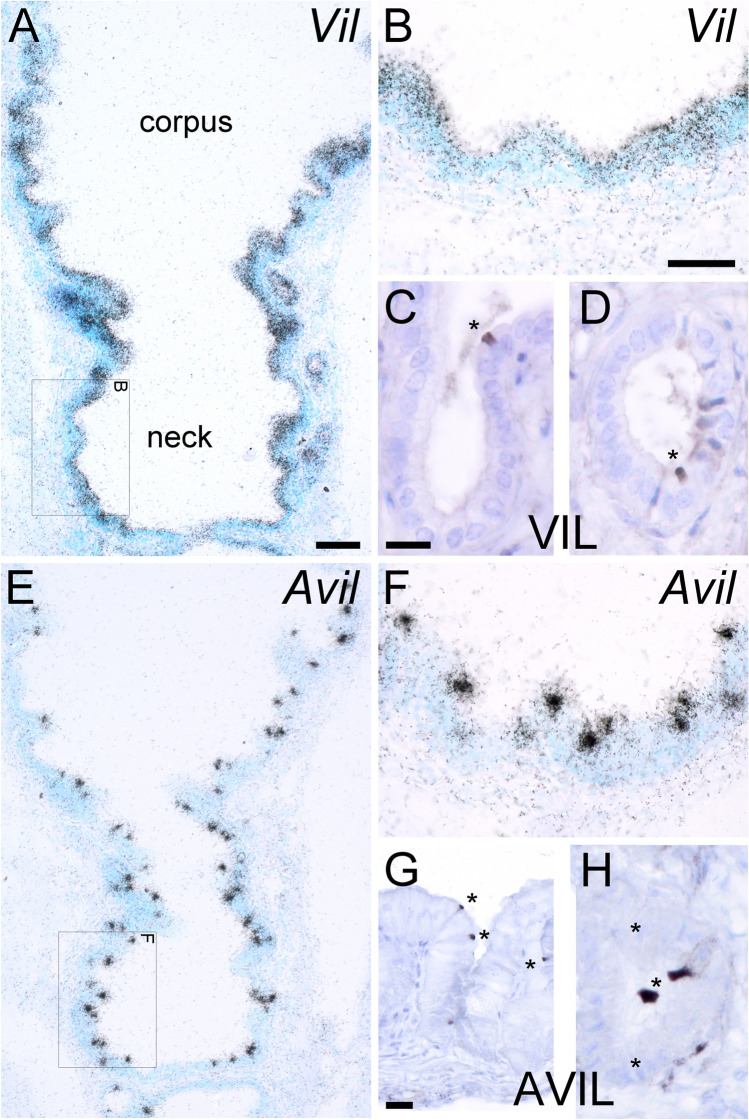
Villin and advillin expression patterns in the mouse gall bladder. (**a**) *Vil* mRNA was present in the entire epithelial lining of the gall bladder, showing no regional accumulations e.g. in neck or corpus. (**b**) higher magnification view from the boxed area in **a**. (**c**) Villin-immunoreactivity was prominent in the apical tips of individual cells in the gall bladder main epithelium (asterisk), and (**d**) in associated glands (asterisk). (**e**) *Avil* mRNA was detected in individual cells in all parts of the gall bladder. (**f**) Higher magnification view from the boxed area in **e**. Advillin-immunoreactivity also was seen in individual cells along the inner lining of the gall bladder, both in the main epithelium (**g**) and in glands (**h**). Sections from ISH were counter-stained with methyl green, sections from IHC with hemalaun. The bar in **a** represents 100 µm and also applies to **e**. The bar in **b** represents 50 µm and also applies to **f**. The bar in **c** represents 10 µm and also applies to **d** and **h**. The bar in **g** represents 20 µm. (Color figure online)

The mouse gall bladder and extrahepatic bile duct epithelia harbor high densities of tuft cells (Iseki 1991; Luciano and Reale 1969; Schütz et al. 2015). We found that *Vil* ISH labeled the entire epithelium of the gall bladder (Fig. 4a, b), while *Avil* mRNA was expressed by solitary cells (Fig. 4e, f). On the protein level, immunoreactivity for villin was detectable in the apical tips of individual cells (Fig. 4c, d), both in the main epithelium and in peribiliary glands. A similar staining pattern was observed with anti-advillin antibodies (Fig. 4g, h). Taken together, while villin was found expressed by almost all cholangiocytes and tuft cells of the gall bladder, advillin expression was found restricted to individual cells, both on the mRNA and on the protein level, suggesting selective presence in tuft cells.

### Advillin and villin co-expression patterns

**Fig. 5:**
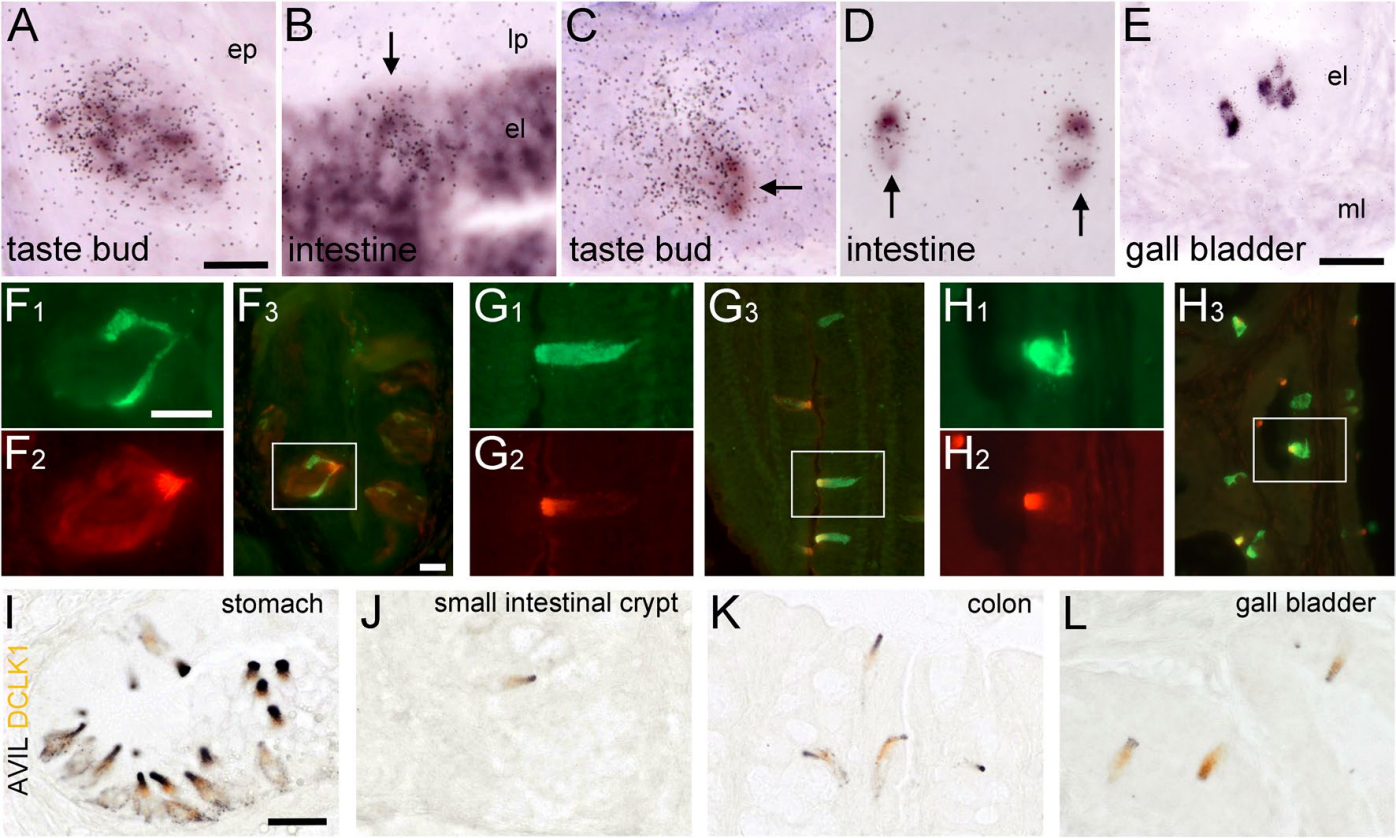
Double-ISH and double-IHC experiments. Simultaneous detection of *Vil* (non-radioactive label) and *Avil* (radioactive label) mRNAs in a mouse taste bud (**a**), and in the duodenum (**b**). Note that *Avil* signals overlap with *Vil* (arrow in **b**). Simultaneous detection of *Chat-Egfp* (non-radioactive label) and *Avil* (radioactive label) mRNAs in a mouse taste bud (**c**), in the duodenum (**d**), and in the gall bladder (**e**). Note partial overlap in taste bud (arrow in **c**) and complete overlap of both labels in intestine (arrows) and in gall bladder. Double-IHC analysis of ChAT-EGFP (green label) with advillin (red label) in taste buds (**f**), in the duodenum (**g**), and in the gall bladder (**h**). The boxed area in the composite pictures f3, g3, and h3 is presented in magnification with the single label in f1, 2, g1, 2, and h1, 2. Double brightfield IHC analysis of advillin (black reaction product) with DCLK1 (brown reaction product) at the squamo-columnar junction of the stomach (**i**), in the small intestine (**j**), in the large intestine (**k**), and in the gall bladder (**l**). *el* epithelial layer; *ep* epidermis; *lp* lamina propria; *ml* muscle layer. The bar in **a** represents 20 µm and also applies to **b**–**d**. The bar in **e** represents 25 µm. The bar in **f1** represents 25 µm and also applies to **f2**, **g1, 2**, **h1, 2**. The bar in **f3** represents 20 µm and also applies to **g3** and **h3**. The bar in **i** represents 20 µm and also applies to **j**–**l**. (Color figure online)

To investigate if advillin and villin are co-expressed in epithelial cells we performed double-ISH experiments. Non-radioactive labeling of *Vil* mRNA, in combination with radioactive labeling of *Avil* mRNA resulted in a fully overlapping pattern in vallate papilla taste buds (Fig. 5a). In the small intestine, exemplified here for the duodenum, solitary *Avil* signals co-localized with *Vil* signals in the mucosa (Fig. 5b).

### Advillin expression is confined to tuft cells

Although tuft cells display region-specific heterogeneity in their molecular signatures (Nadjsombati et al. 2018), a core gene signature has emerged that includes expression of *Chat* (O'Leary et al. 2018; Schütz et al. 2015, 2019). To determine if the solitary intestinal and biliary cells expressing advillin message and protein were tuft cells, we utilized a transgenic reporter mouse line that expresses *Egfp* under the control of the *ChAT* promoter (Schütz et al. 2008; von Engelhardt et al. 2007), and performed double-ISH and -IHC experiments to simultaneously detect EGFP and advillin. In taste buds of the vallate papilla, *Egfp* expressing taste cells were visualized by the non-radioactive label (Fig. 5c). Signals representing *Avil* expression covered *Egfp*-expressing and non-expressing cells, indicative of *Avil* expression in both, type II (bitter, sweet, and umami taste) and III (sour and salty taste) taste cells. A similar staining pattern was seen with IHC (Fig. 5f). In the epithelium of the small intestine, *Egfp* and *Avil* expression almost fully overlapped (Fig. 5d, *n* = 3 mice, ≥ 100 cells analyzed). Only occasionally, epithelial cells displaying *Avil*, but not *Egfp* expression, were found in crypts (data not shown). Double-IHC revealed a 91.6 ± 4.7% co-expression frequency along the gastro-intestinal tract (83.3% in stomach, *n* = 66 cells analyzed; 90.8% in duodenum, *n* = 736 cells; 91.8% in jejunum, *n* = 256 cells; 96.9% in ileum, *n* = 350 cells; 95.2% in colon, *n* = 456 cells). In the gall bladder, *Egfp* expressing tuft cells showed almost full co-expression of *Avil* (*n* ≥ 100 cells analyzed) in double-ISH experiments (Fig. 5e), and 88.6% co-expression frequency in double-IHC (*n* = 184 cells analyzed, Fig. 5h). All other non-co-expressing immunoreactive cells along the gastro-intestinal and biliary tracts had an advillin^+^/EGFP^−^ phenotype. DCLK1 is another established tuft cell marker, and also present in rather immature tuft cells of intestinal crypts (Bjerknes et al. 2012; Gerbe et al. 2011). Co-staining for advillin and DCLK1 in brightfield IHC revealed a complete overlap at the limiting ridge of the stomach (Fig. 5i), in small intestinal crypts (Fig. 5j), large intestine (Fig. 5k), and gall bladder (Fig. 5l) (> 100 cells analyzed per tissue), suggesting that, especially in the intestine, advillin is expressed during all stages of tuft cell maturation.

### Advillin expression pattern in the absence of tuft cells

**Fig. 6:**
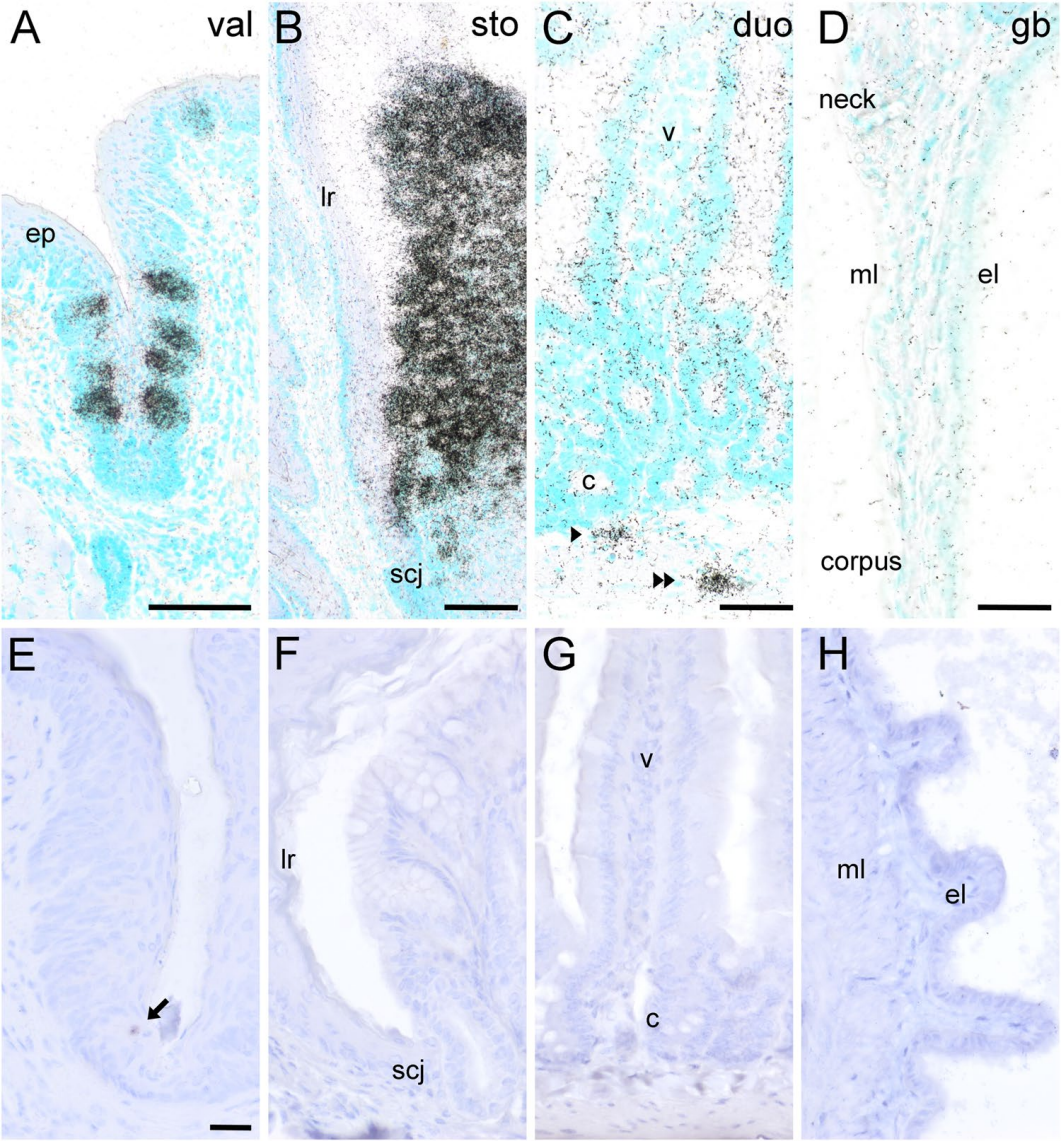
Advillin expression pattern in *Pou2f3*^−/−^ mice. *Avil* mRNA, detected with antisense riboprobes in radioactive ISH, was present in vallate papilla taste buds (**a**), and in the epithelium of the glandular stomach (**b**) of *Pou2f3*^−/−^ mice. *Avil* mRNA was absent from intestinal crypts and villi, but present in some neurons of the submucous (arrowhead) and myenteric (doubled arrowhead) plexus (**c**). *Avil* mRNA was absent from the gall bladder epithelium (**d**). Using IHC, advillin-immunoreactivity was detected faintly in taste buds (**e**, arrow), but was absent from the epithelium of stomach (**f**), duodenum (**g**), and gall bladder (**h**). Sections from ISH were counter-stained with methyl green, sections from IHC with hemalaun. *c* crypt; *el* epithelial layer; *ep* epidermis; *lr* limiting ridge; ml, muscle layer; *v* villus. The bars in **a** and **b** represent 100 µm. The bars in **c** and **d** represent 50 µm. The bar in **e** represents 20 µm and also applies to **f**–**h**. (Color figure online)

Gastro-intestinal tuft cells have been ascribed chemosensory, immunomodulating, and neuromodulating functions (O'Leary et al. 2018). However, it is still under debate if a single type of tuft cell exerts all these functions, or if several tuft cell lineages exist. Since not all cholinergic tuft cells co-expressed advillin in our double-labeling immunohistochemical experiments (see above), we asked whether the additional single-labeled advillin cells represented a different tuft cell type, e.g. non-chemosensory tuft cells, or even enteroendocrine cells (Sutherland et al. 2007). Thus, we analyzed by ISH tissue from *Pou2f3*^*−/−*^ mice that lack type II taste cells (Matsumoto et al. 2011) and chemosensory-type, TRPM5-positive tuft cells (Matsumoto et al. 2011; Yamashita et al. 2017). *Avil* mRNA was readily detected in the vallate papilla taste buds (Fig. 6a). In the stomach, almost all epithelial cells of the glandular stomach at the squamo-columnar junction (Fig. 6b) still gave *Avil* ISH signals. In the small intestine (exemplified here for duodenum), however, *Avil* ISH signals were completely absent from villi and crypts, while ISH signals were still detected in some neurons in the submucous and myenteric plexus (Fig. 6c). *Avil* ISH signals were also completely absent from the gall bladder epithelium (Fig. 6d). In comparison, advillin staining by IHC was faintly present in vallate papilla taste buds (Fig. 6e), but absent from all epithelial cells along the gastro-intestinal and biliary tract (Fig. 6f–h).

### Advillin promoter-driven expression of Cre-recombinase in tuft cells

**Fig. 7:**
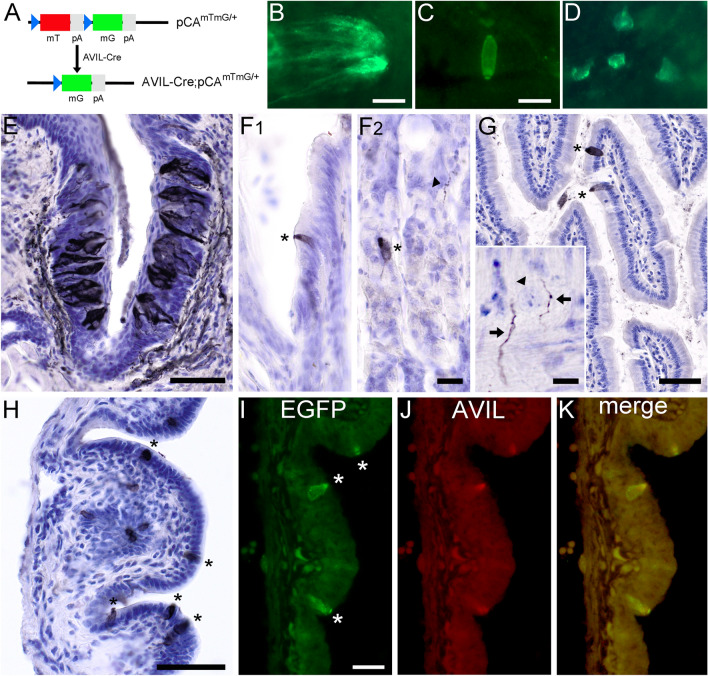
*Avil*-Cre mice are a tool to genetically target tuft cells in the mouse alimentary tract. The mating of mice that harbor the double fluorescent reporter, mT/mG, with a mouse line that expressed Cre-recombinase under the control of the advillin promoter (*Avil*-Cre) resulted in cell-specific excision of *mT* due to flanking *loxP* (blue triangle) sites, and activation of *Egfp* expression (**a**). pA = polyadenylation site. Native EGFP fluorescence in fresh tissue representing Cre activity in vallate papilla (**b**), duodenal mucosa (**c**), and gall bladder (**d**). Enhancing EGFP detection with antibodies revealed presence of EGFP in vallate papilla taste buds, and in innervating presumably sensory nerve fibers (**e**). Similarly, EGFP was present in solitary cells at the limiting ridge (**f1**, marked by asterisk) and in the glandular corpus of the stomach (**f2**, marked by asterisk), and in nerve fibers (arrowhead in **f2**) traveling bottom-up in these glands. In the duodenum, solitary cells in the mucosal epithelium were labeled (asterisks) in addition to presumably sensory nerve fibers in the muscle layers (arrows in inset), while nerve cell bodies were not stained (arrowhead in inset) (**g**). EGFP-positive cells were present in the epithelium of the gall bladder (asterisks) (**h**). Double-immunofluorescence analysis of EGFP (**i**, green label) and advillin (**k**, red label) showed co-existence (**k**) in the duodenum. The bar in **b** represents 10 µm and also applies to **d**. The bar in **c** represents 20 µm. The bars in **e**, **g** (inset 10 µm), and **h** represent 50 µm. The bar in **f** represents 20 µm. The bar in **i** represents 20 µm and also applies to **j**, **k**. (Color figure online)

In our present study advillin was found to be a tuft cell-specific protein, especially in the mucosal epithelia of the intestine and gall bladder. Thus, we wanted to know if the *Avil* promoter could serve as a genetic tool to specifically target intestinal and biliary tuft cells. Hemizygous male *Avil*-Cre mice were mated with homozygous female mT/mG mice. The double-fluorescent reporter mice carry a cDNA for a red fluorescent (tdTomato*)* protein that is flanked by *loxP* sites, followed by a polyadenylation sequence and the *Egfp* coding sequence. The *tdTomato* gene will be selectively removed in the presence of active Cre-expression, and instead the *Egfp* gene transcribed (Fig. 7a). In progeny from these matings, native EGFP fluorescence was detectable in taste cells of the vallate papilla (Fig. 7b), and in solitary epithelial cells of both, the intestine (Fig. 7c) and the gall bladder (Fig. 7d). Enhancing EGFP detection with anti-EGFP antibodies resulted in a strong labeling of cells in vallate papilla taste buds and innervating sensory nerve fibers (Fig. 7e). Single EGFP-immunoreactive cells lined the columnar epithelium at the gastric groove and were also present in the glandular stomach (Fig. 7f). In the intestine, solitary EGFP-immunoreactive epithelial cells were detected in villi and crypts, in addition to a few presumably sensory nerve fibers coursing through the muscle layers (Fig. 7g). Gastro-intestinal nerve cell bodies were devoid of detectable EGFP expression (inset in Fig. 7g). Finally, many EGFP-immunoreactive cells lined the epithelium of the gall bladder (Fig. 7h). Double immunofluorescence with EGFP and advillin antibodies (Fig. 7i–k) revealed that recombination events had been obtained in true advillin expressing tuft cells. To obtain information about the targeting-efficiency of the *Avil*-Cre promoter we determined the EGFP/DCLK1 co-expression pattern in these mice. At the limiting ridge of the stomach, 58.6 ± 28.7% of the DCLK1-immunoreactive tuft cells were traced. In duodenum (15.6 ± 5.5%), jejunum (12.2 ± 5.3%), ileum (18.0 ± 6.2%), and colon (33.4 ± 8.6%) low targeting frequencies were obtained. Finally, in the gall bladder 99.0 ± 1.8% of the DCLK1 tuft cells were also EGFP-positive. These data are proving a tissue- and region-dependent applicability of *Avil*-Cre mice to genetically target gastro-intestinal and biliary tuft cells.

## Discussion

The introduction of villin-antibodies as tuft cell marker in immunohistochemical analysis dates back almost 30 years (Höfer and Drenckhahn 1992). In mice, a comparison of the transcriptional profiles of small intestinal TRPM5-positive tuft cells with that of other enterocytes revealed that villin was expressed equally strong in both cell populations, while advillin expression was restricted to tuft cells (Bezençon et al. 2008). To be sure that our immunohistochemical tools had an adequate specificity, we stained sections containing taste cells, which express both, villin and advillin, and dorsal root ganglia that are known to express advillin but not villin (Chuang et al. 2018). We found similar staining patterns for villin and advillin in taste cells, however many dorsal root ganglia neurons stained for advillin, but not for villin, thus ruling out advillin cross-reactivity of the anti-villin antibody. Importantly, the anti-advillin antibody we used in our study did not stain the entire intestinal surface epithelium, as seen with anti-villin antibodies, which further validated their specificity. Hence, both immunohistochemical tools we employed showed an appropriate selectivity in detecting their cognate proteins.

We here documented the advillin expression pattern in the mouse alimentary tract on a cellular histological level. The major finding of our study is that advillin, unlike villin, displays a tuft cell-restricted expression pattern in the intestinal and biliary tract mucosal epithelia, both on the mRNA and protein levels. In addition, we found that advillin expression on the protein level was completely absent from the gastro-intestinal and biliary tract mucosa of *Pou2f3*^−/−^ mice, while expression was still present in oral taste cells, and on the mRNA level in stomach. This suggests that advillin is also expressed in type III taste cells that detect sour and salty taste modalities, and that extra-oral expression is confined to the chemosensory tuft cell lineage, and to sensory neurons. In our double-labeling immunohistochemical experiments we observed that about 10% of the advillin-immunoreactive cells did not co-express EGFP driven by the *Chat* promoter. On the other hand, a completely overlapping expression pattern was seen with another tuft cell marker, DCLK1. This suggests that advillin is expressed early on in the tuft cell lineage, while the cholinergic phenotype of these cells may be established at a more later time point of cell maturation. Hence, we propose that advillin should be added as a morphology marker to the tuft cell minimal gene signature, which already includes POU2F3, IL-25, COX1 and COX2, ALOX5, TRPM5, and ChAT (reviewed by O'Leary et al. 2018). Our actual findings in mice are underscored by our recent analysis of the distribution pattern and molecular signature of cholinergic tuft cells in the normal human alimentary tract. There, presence of advillin-immunoreactivity was also confined to ChAT-positive tuft cells in the small and large intestine, in peribiliary glands, and in small intra- and interlobular pancreatic ducts (Schütz et al. 2019).

A discrepancy between the tissue distribution pattern of mRNA versus protein was observed for advillin in the stomach. Messages were present in seemingly all epithelial cells, even in *Pou2f3*^−/−^ mice, while detectable protein expression was confined to solitary tuft cells in immunohistochemical analysis. First, this could mean that all epithelial cells in the stomach produce advillin, with non-tuft cells expressing this protein at levels that are below the detection limit in immunohistochemical experiments. Second, lack of protein detection in all but tuft cells may result from a post-transcriptional silencing mechanism. Third, a cross-reactivity of our *Avil*-mRNA ISH probe with another expressed gene may occur in this organ. Although the definite reason for the observed discrepancy remains enigmatic, our finding of a restriction of *Avil*-promoter-driven Cre recombinase activity to solitary cholinergic tuft cells in the entire gastro-intestinal and biliary tract strongly suggests that functionally relevant *Avil* gene expression and advillin quantities are only found in tuft cells. A similar discrepancy between the presence of mRNA and protein was observed for villin in stomach and biliary tract, while detection completely matched in the intestine. Again, functionally relevant protein amounts may differ along the aspects of the alimentary and biliary tracts.

Originally, advillin expression was considered to be largely restricted to sensory neurons (Chuang et al. 2018). We were able to show by IHC that advillin was present in sensory neurons of dorsal root ganglia, while villin was not. Advillin mRNA was also detected by us in neurons of the intestinal submucosal and myenteric plexus. This would be in agreement with a recent report stating that advillin is present in all neurons of the autonomic nervous system (Hunter et al. 2018). However, gut-intrinsic neurons never stained positive in our own IHC using anti-advillin antibodies, nor did they display EGFP-immunoreactivity in our analysis of an *Avil*-Cre:mT/mG double transgenic mouse line. Instead, presumably sensory nerves were prominent around taste buds in vallate papillae, in the muscle layers of the intestine, and in the glandular stomach of *Avil*-Cre:mT/mG mice. This indicates that *Avil*-promoter driven Cre expression and activity is not established in gut autonomic plexus neurons in the *Avil*-Cre mouse line we used. Recent support comes from our targeting of tracheal brush cells, where a conditional elimination of brush cell-derived ACh, but not neuronal ACh, was obtained by crossing the *Avil-*Cre mouse line with mice that harbor a floxed ChAT allele (Misgeld et al. 2002). Trachea preparations from these mice still retained neural cholinergic signaling, i.e. intact cholinergic constriction evoked by electrical field stimulation. The effect of a formylated bacterial peptide on stimulating particle transport speed, however, was almost fully lost (Perniss et al. 2020).

Tuft cell biology has recently received tremendous attention since the discovery that they may function as chemosensory sentinels that monitor intestinal, respiratory, and urethral infection, and that they initiate adequate protective responses through activation of type 2 immunity (recently reviewed by von Moltke and Pepper 2018). Hence, deciphering the specific roles of individual tuft cell components, i.e. receptors and signaling molecules, in these scenarios is warranted to understand in detail how these cells do their job in a given surrounding and pathophysiological situation. Uncovering the multiple functions tuft cell components play can be achieved by tuft cell-specific manipulation of gene expression. Along this line, multiple transgenic mouse tools have been implemented, that are more or less tuft cell-specific. Currently available mouse lines that express Cre-recombinase in tuft cells are driven by *Pou2f3* (McGinty et al. 2020), *Trpm5* (Kusumakshi et al. 2015), *Dclk1*, *Vil*, *Chat*, *Tas2r131*, or *Tas2r143* promoters (recently summarized by O'Leary et al. 2018). Our own evaluation of a mouse line that expresses Cre-recombinase under the control of the advillin promotor revealed region-selective efficiency differences in targeting tuft cells. Recombination rates were highest in the gall bladder, and rather low in stomach, small and large intestine. Hence, the applicability of the *Avil*-Cre mouse line to genetically target tuft cells must be critically reflected before time-consuming experiments are planned and performed. At least biliary tuft cells, and the related tracheal brush cell (see above) resemble valuable and promising targets. Nevertheless, we now propose that *Avil*-Cre mice can be added to this armory and may serve as a versatile tool to genetically target primarily biliary tuft cells in the mouse alimentary tract. The availability of an inducible variant of *Avil*-Cre, i.e. the *Avil-CreERT* mouse line (Hunter et al. 2018; Lau et al. 2011), would even allow genetic manipulation of tuft cell components on demand.

## Electronic supplementary material

Below is the link to the electronic supplementary material.

Electronic supplementary material 1 (PNG 2007 kb)

Electronic supplementary material 2 (PNG 1022 kb)

Electronic supplementary material 3 (PNG 1325 kb)

Electronic supplementary material 4 (PNG 1346 kb)

Electronic supplementary material 5 (PNG 843 kb)

Electronic supplementary material 6 (PNG 1200 kb)

Electronic supplementary material 7 (PNG 1216 kb)
