# Supplementary figures and images for: Advillin is a tuft cell marker in the mouse alimentary tract
Source: J Mol Histol. 2020 Jul 2;51(4):421–35. doi: 10.1007/s10735-020-09893-6 (PMC7368872; doi:10.1007/s10735-020-09893-6)

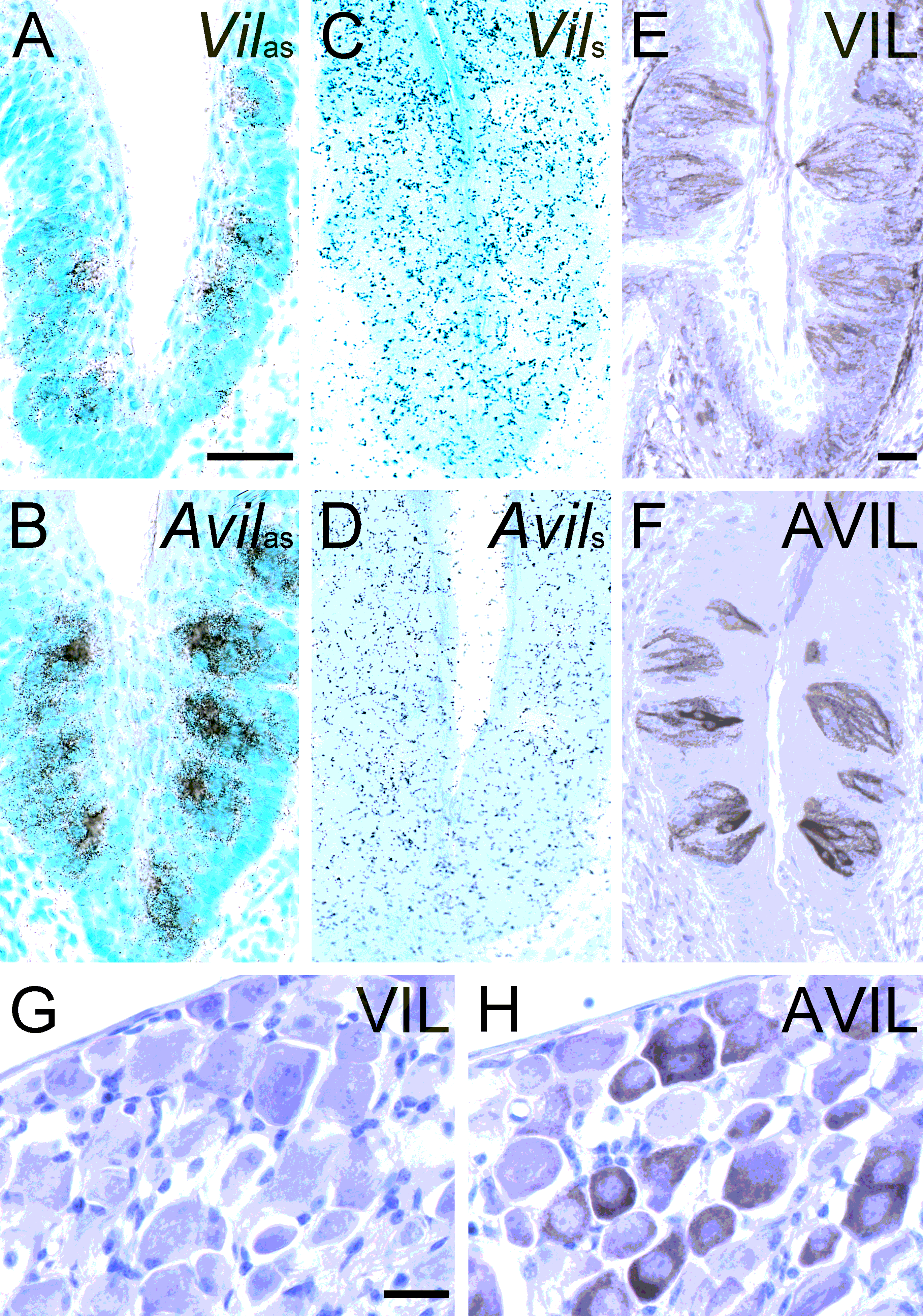

Supplement: Supplementary file 1 — Electronic supplementary material 1 (PNG 2007 kb) [file 10735_2020_9893_MOESM1_ESM.png]

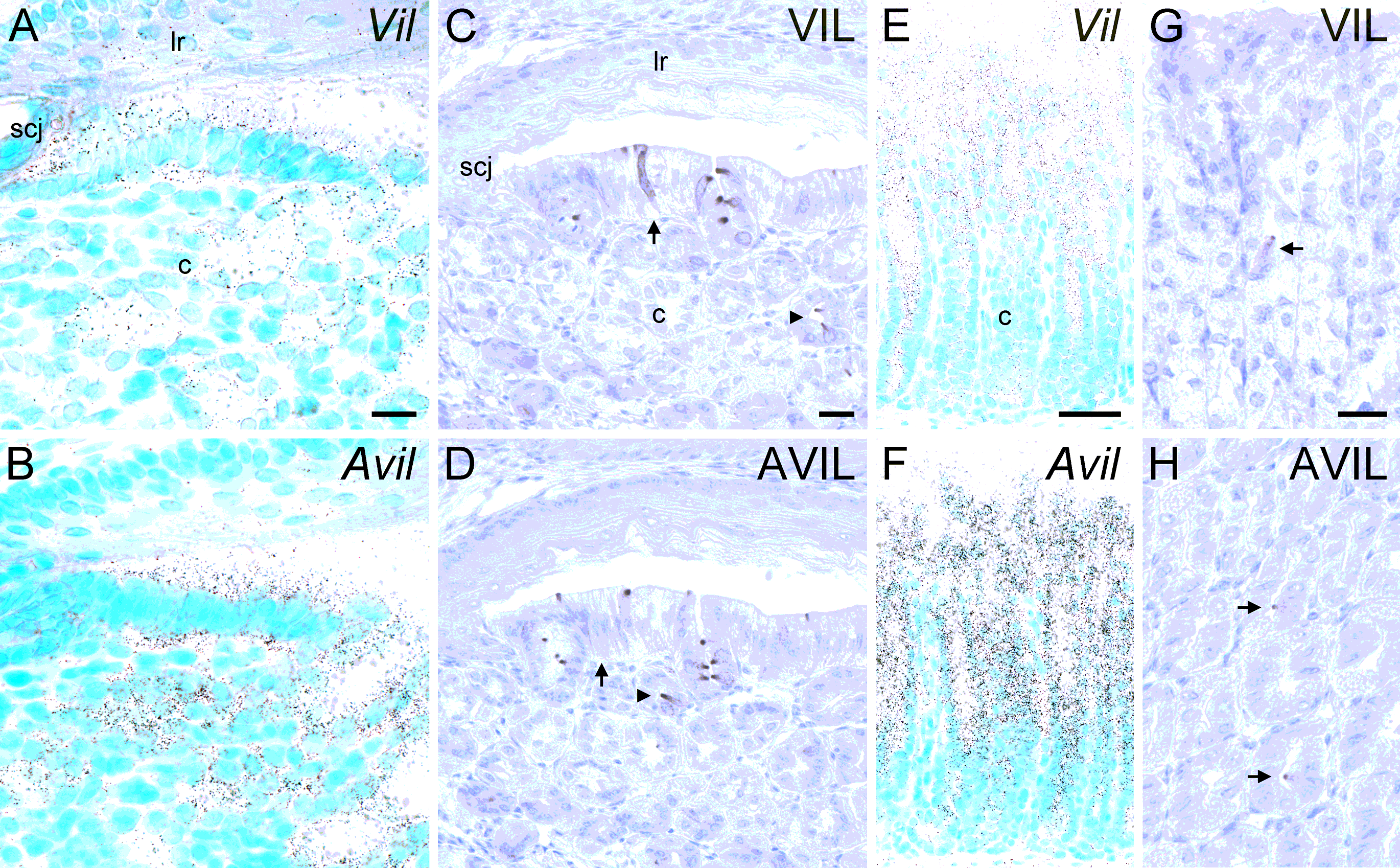

Supplement: Supplementary file 2 — Electronic supplementary material 2 (PNG 1022 kb) [file 10735_2020_9893_MOESM2_ESM.png]

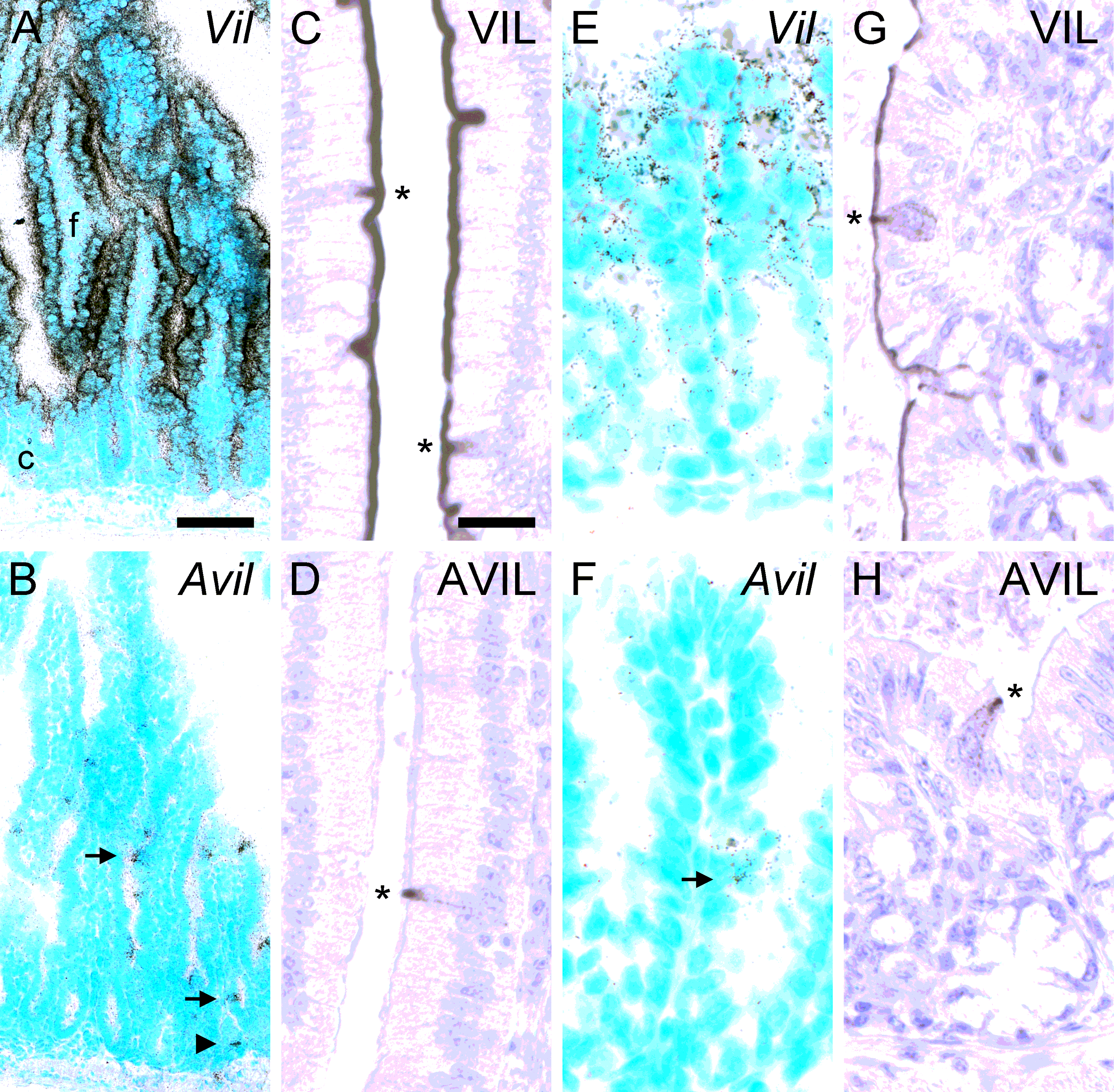

Supplement: Supplementary file 3 — Electronic supplementary material 3 (PNG 1325 kb) [file 10735_2020_9893_MOESM3_ESM.png]

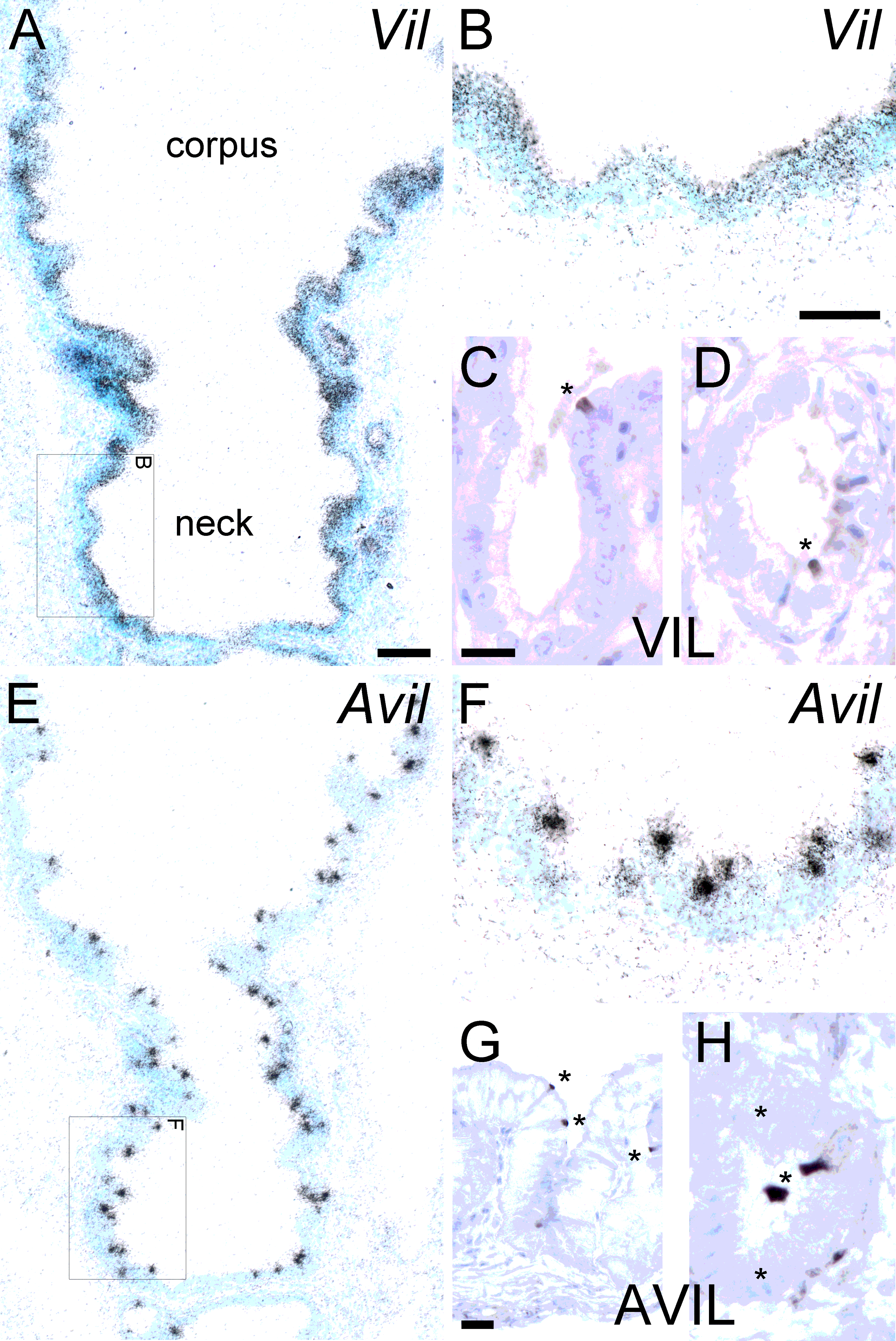

Supplement: Supplementary file 4 — Electronic supplementary material 4 (PNG 1346 kb) [file 10735_2020_9893_MOESM4_ESM.png]

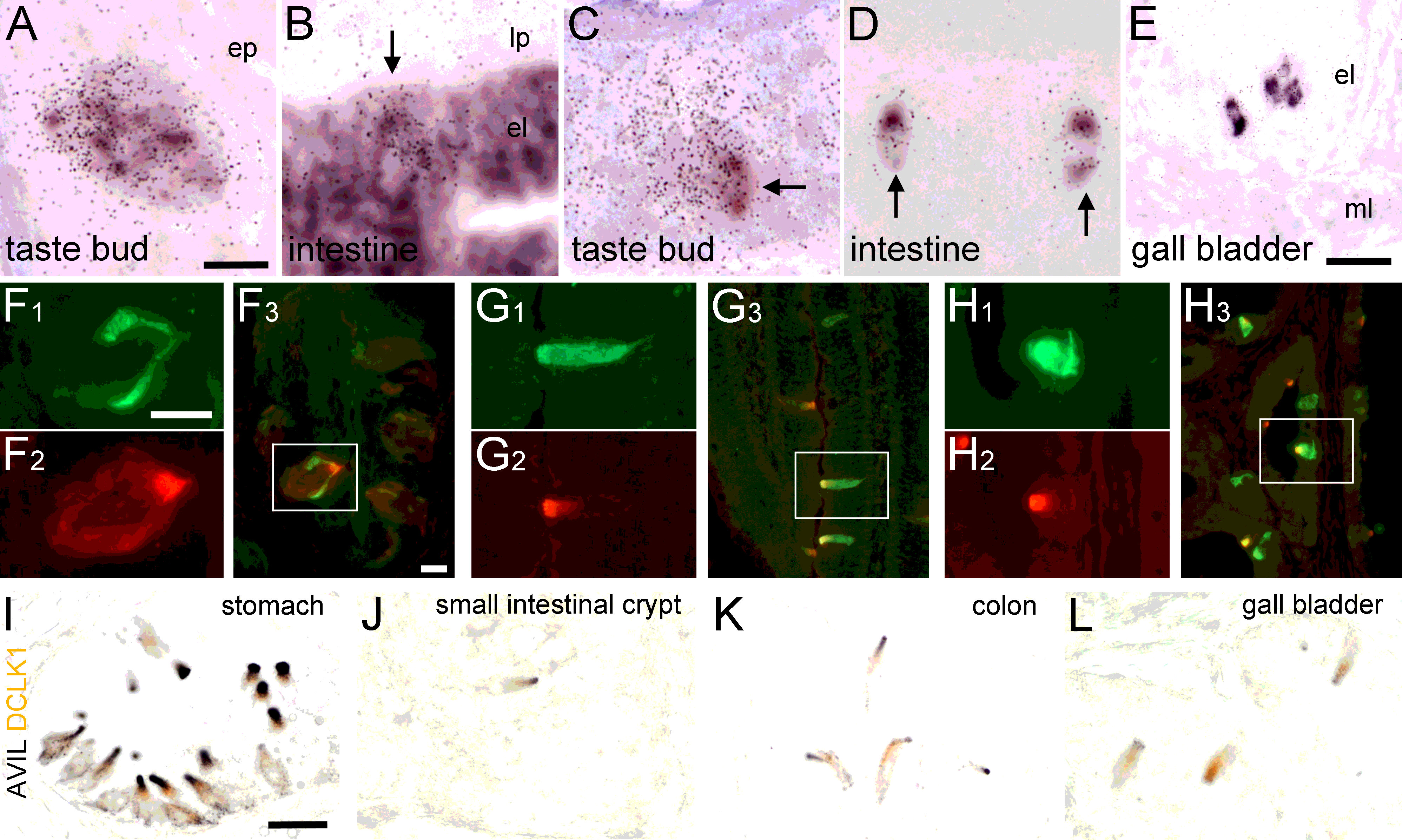

Supplement: Supplementary file 5 — Electronic supplementary material 5 (PNG 843 kb) [file 10735_2020_9893_MOESM5_ESM.png]

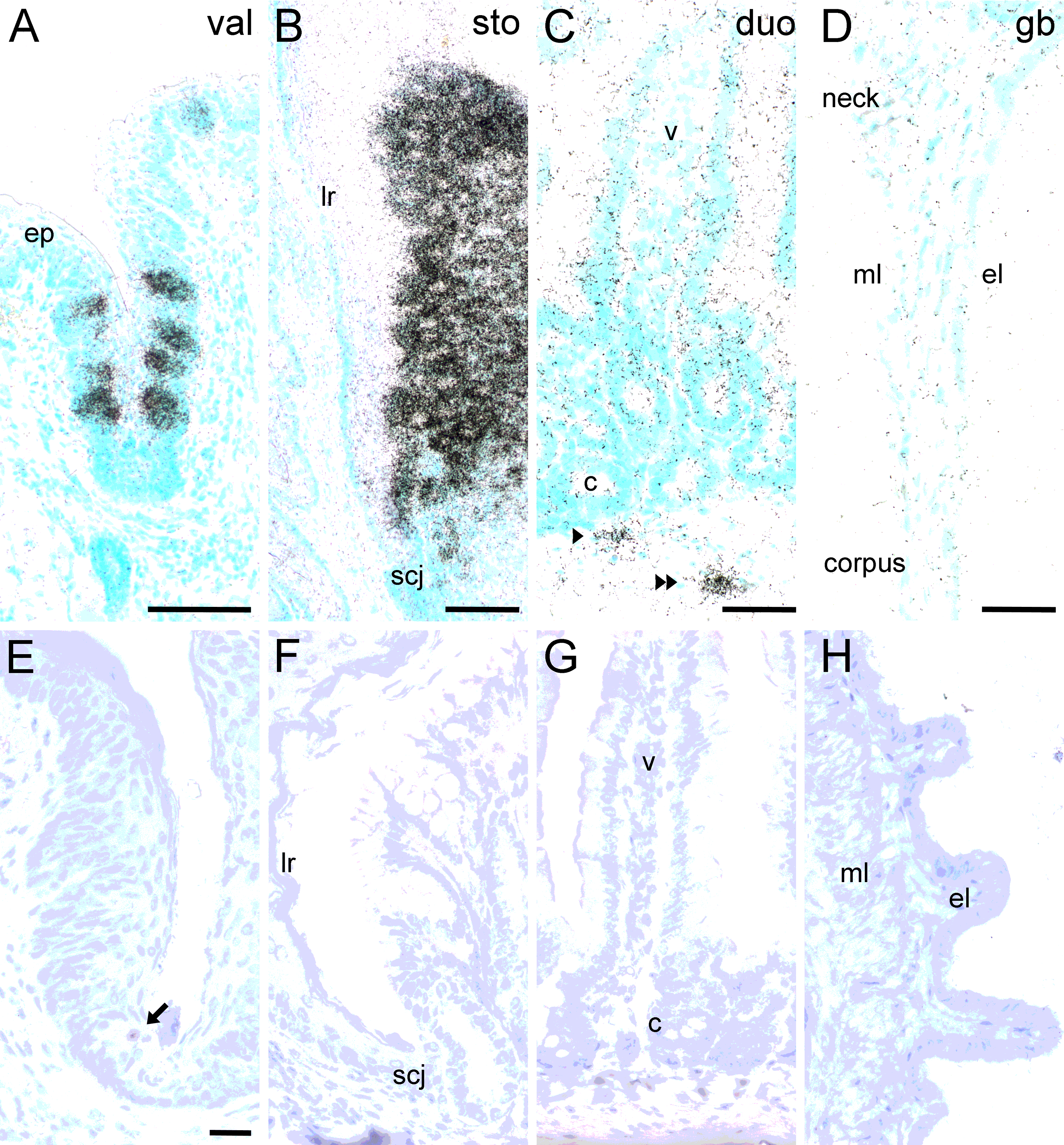

Supplement: Supplementary file 6 — Electronic supplementary material 6 (PNG 1200 kb) [file 10735_2020_9893_MOESM6_ESM.png]

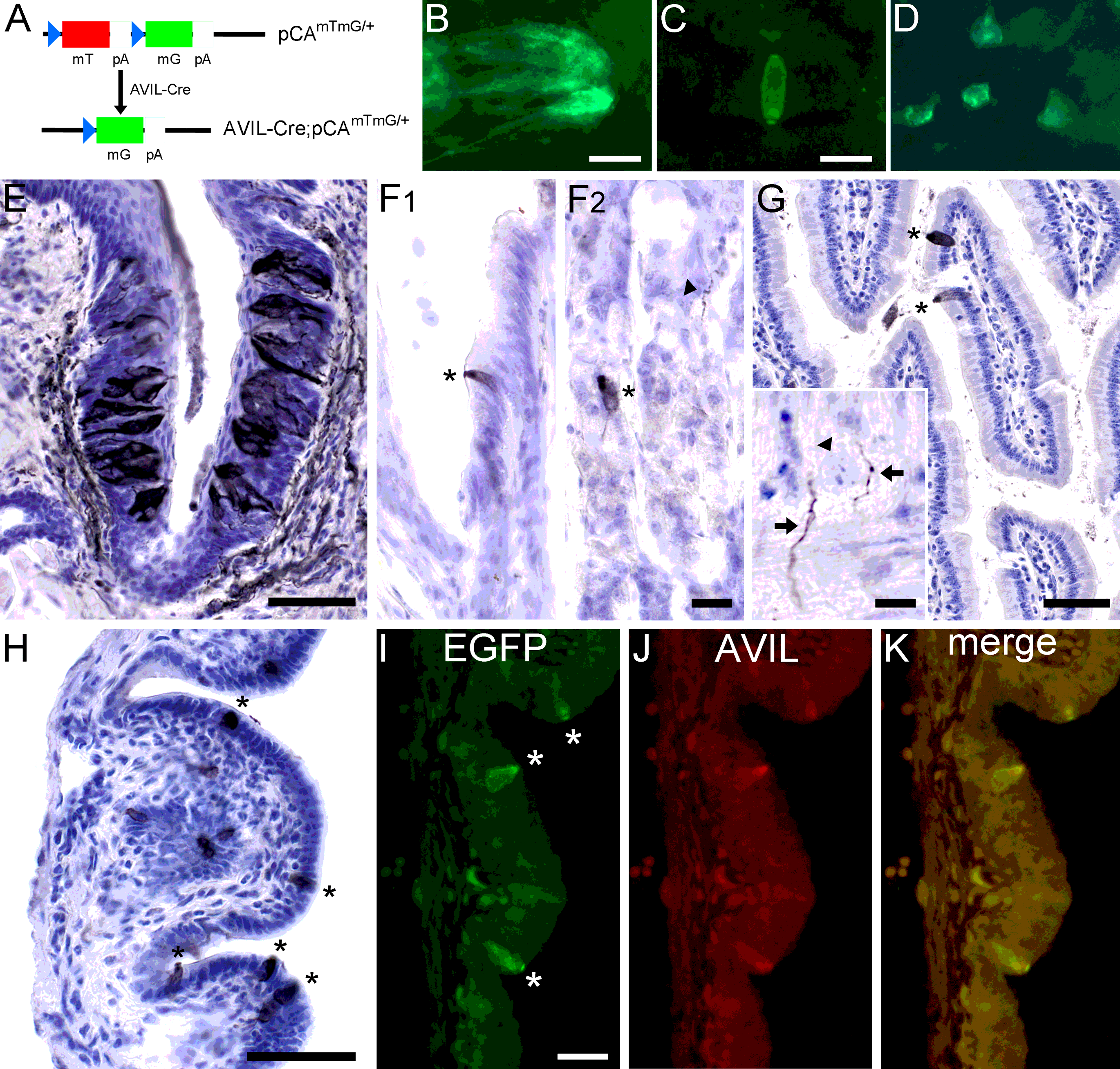

Supplement: Supplementary file 7 — Electronic supplementary material 7 (PNG 1216 kb) [file 10735_2020_9893_MOESM7_ESM.png]
